# Topological features of brain functional networks are reorganized during chronic tinnitus: A graph‐theoretical study

**DOI:** 10.1111/ejn.16643

**Published:** 2025-01-13

**Authors:** Shuting Han, Yongcong Shen, Xiaojuan Wu, Hui Dai, Yonggang Li, Jisheng Liu, Duo‐duo Tao

**Affiliations:** ^1^ Department of Radiology the First Affiliated Hospital of Soochow University Suzhou China; ^2^ Department of Ear, Nose, and Throat The First Affiliated of Soochow University Suzhou China

**Keywords:** functional neuroimaging, network reorganization, neuroplasticity, topographic connections

## Abstract

This study aimed to investigate the topological properties of brain functional networks in patients with tinnitus of varying durations. A total of 51 tinnitus patients (divided into recent‐onset tinnitus (ROT) and persistent tinnitus (PT) groups) and 27 healthy controls (HC) were recruited. All participants underwent resting‐state functional MRI and audiological assessments. Graph theory was used to examine brain network topology.

The results showed that the ROT group exhibited lower clustering coefficient, gamma, sigma and local efficiency compared to both the HC and PT groups (all *P* < 0.05). Significant reductions in nodal clustering coefficient and local efficiency were found in the left caudate nucleus and left olfactory cortex, while increased nodal centralities were observed in the left orbital middle frontal gyrus and left postcentral gyrus in ROT patients (all *P* < 0.05). Furthermore, the ROT group had decreased nodal clustering in the right lenticular putamen and reduced nodal efficiency in the left olfactory cortex compared to both PT patients and HCs (all *P* < 0.05).

Additionally, PT patients showed weaker functional connectivity between the subcortical and occipital lobe modules, as well as between the prefrontal and intra‐frontal modules, compared to ROT patients. However, intra‐module connectivity in the subcortical module was stronger in PT patients than in HCs.

These findings suggest that recent‐onset tinnitus is associated with alterations in brain network topology, but many of these changes are restored with the persistence of tinnitus.

## Graphical abstract

The flow chart illustrates the study design, where tinnitus patients are categorized into recent‐onset (ROT) and persistent (PT) groups, with healthy controls (HC) recruited. Participants underwent resting‐state fMRI and tinnitus related audiological assessments. Graph theory analysis was then applied to metrics such as cluster coefficient, network efficiency, and the strength of inter‐modular and intra‐modular connections to identify group differences and alterations in brain network topology related to tinnitus duration.

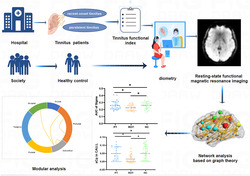

**Abbreviations**
3D‐MPRAGE: Three‐dimensional magnetization‐prepared rapid acquisition gradient echo sequence
ANCOVA: Analysis of covariance
AUC: Area under the curve
BC: Betweenness centrality
BOLD: Blood‐oxygen‐level‐dependent
Cp: Clustering coefficient
DC: Degree centrality
DPABI: Data processing & Analysis Imaging
Eglob: Global network efficiency
Eloc: Local network efficiency
EPI: Echo Planar Imaging
fALFF: Fractional amplitude of low‐frequency fluctuation
FOV: Field of view
GRF: Gaussian random field
HC: Healthy controls
KCC: Kendall coefficient of Concordance
L: Left
LM: Loudness matching
Lp: Characteristic path length
LSD: Least significant difference
MNI: Montreal Neurological Institute
NA: Not applicable
NCp: Nodal clustering coefficient
Ne: Nodal efficiency
Nle: Nodal local efficiency
NLp: Nodal characteristic path length
ORBmid.L: Orbital part of left middle frontal gyrus
PM: Pitch matching
PT: Persistent tinnitus
ROI: Region of interest
RET: Recent‐onset tinnitus
rs‐fMRI: Resting‐state functional magnetic imaging
SD: Standard deviation
TE: Echo time
TFI: Tinnitus Functional Index
TR: Repetition time

## 1 INTRODUCTION

Tinnitus is the perception of sound in the absence of any external acoustic stimuli or internal sources of auditory perception, with a global prevalence ranging from 11.9% to 30.3% (McCormack et al., 2016). Patients with tinnitus experience not only auditory symptoms, such as hyperacusis and hearing difficulties, but also psychological symptoms, including anxiety, depression, insomnia and inattention. These symptoms are closely related to the severity of tinnitus (Langguth, 2011). According to the *US Clinical Practice Guidelines: Tinnitus*, published in 2014, tinnitus is classified as recent‐onset tinnitus (duration <6 months) and persistent tinnitus (duration ≥6 months) (Tunkel et al., 2014). Recent‐onset and persistent tinnitus often exhibit different clinical features, such as variations in loudness, frequency and mood disturbances associated with tinnitus (Wallhäusser‐Franke et al., 2017; Zhang et al., 2023). A recent longitudinal study found that 18.4% of patients with recent‐onset tinnitus experienced complete resolution within 6 months, whereas patients with persistent tinnitus showed no change in tinnitus characteristics (Vielsmeier et al., 2020). However, the precise mechanism underlying the chronicity of tinnitus remains unclear.

The pathophysiology of tinnitus is complex and multifactorial, involving both peripheral and central systems. Tinnitus was initially thought to originate from aberrant neural activity localized to the peripheral auditory system; however, recent studies indicate that tinnitus is associated with abnormal neural activity across widely distributed brain networks. These networks include both the auditory network and extra‐auditory structures, such as the frontal cortex, parahippocampus, cingulate cortex, insula and cerebellum (Chen et al., 2015, 2017; Cheng et al., 2020; De Ridder et al., 2023). This abnormal neural activity spans multiple brain networks (Kok et al., 2022). Several studies have identified increased auditory connectivity in tinnitus patients. Hinkley et al. (2015) observed increased functional connectivity (FC) between the bilateral primary auditory cortex and non‐primary auditory cortex, as did Cai et al. (2019). The precuneus, considered a central hub of the default mode network (DMN), was found by Chen et al. to exhibit increased FC between the anterior cingulate cortex and the left precuneus, correlating with both tinnitus severity and duration (Chen, Chen, et al., 2018; Chen, Liu, et al., 2018; Utevsky et al., 2014). Additionally, the attention network located in the frontoparietal lobe has been shown to have increased functional connectivity in tinnitus patients (Burton et al., 2012; Chen et al., 2015; Job et al., 2020). Xu et al. examined the frontostriatal circuit in tinnitus patients without hearing loss, finding that changes in the limbic system were associated with both the severity and duration of tinnitus (Xu, Cui, et al., 2019).

The majority of these studies utilized seed‐based whole‐brain FC analysis; however, the requirement of selecting specific regions of interest (ROIs) in this analysis method poses challenges for reproducibility. Few studies have consistently selected identical ROIs, thereby limiting reproducibility in the field. When classifying tinnitus by onset duration, numerous studies have focused on developing pathophysiological models for persistent (chronic) tinnitus. However, few studies have explored the neuronal changes that occur in the progression from recent‐onset to persistent tinnitus. In a high‐density electroencephalography study, Lan et al. observed that the transition from acute to chronic tinnitus is accompanied by changes in local brain activity and connectivity between the parahippocampal gyrus and other non‐auditory regions (Lan et al., 2021). Using arterial spin labelling perfusion magnetic resonance imaging, Hu et al. identified that acute and chronic tinnitus patients exhibit different cerebral blood flow changes in the right superior temporal gyrus and frontal gyrus, which correlate with certain tinnitus features (Hu et al., 2021).

The graph theory analysis method can be employed to understand resting‐state functional MRI (rs‐fMRI) connectivity at the whole‐brain level. Lin et al. identified topological network changes, including generally heightened connectivity within the global network and altered auditory‐limbic connections in tinnitus patients (Lin et al., 2020). However, this study utilized structural brain networks constructed based on inter‐regional cortical thickness/subcortical volume correlations and did not explore the relationship between topological properties and tinnitus duration. Lan et al. compared resting‐state brain functional networks in patients with acute and chronic tinnitus with healthy controls, finding that the topological characteristics of the brain network in tinnitus patients were altered in the prefrontal‐limbic‐subcortical regions (Lan et al., 2022). Lan's study correlated graph theory metrics separately with the clinical characteristics of patients with acute tinnitus and those with chronic tinnitus. It is believed that a more comprehensive approach would involve performing correlation analyses between graph theory metrics and the clinical characteristics of all tinnitus patients. Additionally, further assessment could be achieved using different clinical scales, such as the Tinnitus Functional Index, which evaluates the negative impact of tinnitus from multiple perspectives.

In this study, rs‐fMRI data were collected to investigate differences in the topological properties of functional networks across recent‐onset tinnitus, persistent tinnitus and healthy control groups. The graph‐theoretic approach was employed to assess the characteristics of functional integration and segregation in patients with tinnitus at different stages of the disease. We hypothesized that in the early stages of tinnitus, the topological indices of the brain network would experience specific alterations and that as time progresses, the brain would gradually acclimate to or reverse these changes.

## 2 MATERIALS AND METHODS

### 2.1 Subjects

From June 2021 to December 2021, a total of 53 patients with tinnitus were enrolled in the Tinnitus Clinic of the Department of Otolaryngology, The First Affiliated Hospital of Soochow University, and 27 age‐ and gender‐matched healthy controls were recruited. Two patients with tinnitus were excluded due to poor image quality (head displacement greater than 3.0 mm or arbitrary angle rotation greater than 3.0°). A total of 51 patients with tinnitus and 27 healthy controls were included in the study. All participants had at least 12 years of education. Patients with a tinnitus duration of less than 6 months were categorized into the recent‐onset tinnitus group, while those with a duration of 6 months or more were placed in the persistent tinnitus group.

Inclusion criteria for patients with tinnitus were as follows: (1) Tinnitus as the primary complaint. (2) Right‐handed. (3) Ability to independently complete all scale assessments. (4) No use of neurological or psychotropic drugs for at least one month prior to the MRI scan. Exclusion Criteria were as follows: (1) Patients with objective tinnitus. (2) Patients with Meniere's disease, acoustic neuroma, sudden deafness or other inner ear, middle ear or neurological disorders that could cause tinnitus. (3) An average hearing threshold (across 500 Hz, 1000 Hz, 2000 Hz, 4000 Hz) less than 30 dB HL, as measured by pure‐tone audiometry. (4) History of neurological conditions such as major brain trauma, cerebrovascular diseases, brain tumours or neurodegenerative diseases. (5) Psychiatric disorders such as severe anxiety or depression. (6) Contraindications to MRI scanning, such as metal implants or claustrophobia.

Inclusion criteria for healthy controls were as follows: (1) Age and gender matching with tinnitus patients. (2) Right‐handedness. Exclusion Criteria for Healthy Controls were as follows: (1) Average hearing threshold greater than 30 dB HL, as measured by pure‐tone audiometry. (2) History of neurological diseases such as major traumatic brain injury, cerebrovascular disease, brain tumour or neurodegenerative disease. (3) Mental illness, including severe anxiety or depression. (4) Contraindications to MRI scanning, such as metal implants, claustrophobia.

A total of 78 subjects were included in the study, consisting of 28 patients with persistent tinnitus, 23 patients with recent‐onset tinnitus and 27 healthy controls. The assessment and collection of demographic and clinical data (including pure‐tone hearing threshold test, tinnitus psychoacoustic test, and Tinnitus Functional Index) were performed in the same manner for all groups.

### 2.2 Pure‐tone hearing threshold test and tinnitus psychoacoustic characteristics test

All participants were required to undergo a pure‐tone audiometry (PTA) test. Patients with tinnitus were also asked to complete the tinnitus psychoacoustic signature test, which included pitch matching (PM) and loudness matching (LM). These tests were conducted by researchers using ASTERA audiometers in a soundproof room. PTA assesses hearing thresholds at 0.25 kHz, 0.5 kHz, 1 kHz, 2 kHz, 4 kHz and 8 kHz (air conduction) and 0.25 kHz, 0.5 kHz, 1 kHz, 2 kHz and 4 kHz (bone conduction). The average hearing threshold was analysed using the average of the air conduction thresholds across 0.5 kHz, 1 kHz, 2 kHz, and 4 kHz.

The process for testing the psychoacoustic characteristics of tinnitus was as follows: first, the subjects were asked to choose between two sounds of different frequencies until they identified the sound most similar to their own tinnitus. Second, tinnitus loudness matching (LM) was performed by using the ramp‐down method, with the stimulus set 10–15 dB above the threshold of the measured pure‐tone hearing threshold of PM. The subjects listened twice to select the loudness that most closely resembled their subjective tinnitus sound, and the average of the two results was used to enhance the reliability of the test.

### 2.3 Evaluation of Tinnitus Functional Index

All tinnitus patients were also assessed using the Tinnitus Functional Index (TFI), a scale that quantitatively evaluates the severity of tinnitus and its negative effects (Meikle et al., 2012). The TFI consists of 8 subscales with a total of 25 questions. These subscales include Intrusiveness (I), Sense of Control (SC), Cognitive (C), Sleep (SL), Auditory (A), Relaxation (R), Quality of Life (Q) and Emotional (E). Patients were asked to respond to each item on a Likert scale ranging from 0 to 10. The total TFI score ranges from 0 to 100, with higher scores indicating more severe tinnitus and greater negative impact. MRI Data Acquisition.

### 2.4 MRI data acquisition

MRI scans were conducted on a Philips 3.0 T Ingenia MRI scanner equipped with a 15‐channel head coil. During the scans, all participants were instructed to close their eyes and remain awake, using earplugs to reduce scanner noise and foam pads to minimize head movement. T1‐weighted images were acquired using a three‐dimensional magnetization‐prepared rapid acquisition gradient echo sequence (3D‐MPRAGE) with the following parameters: repetition time (TR) = 7.0 ms, echo time (TE) = 3.1 ms, flip angle = 8°, field of view (FOV) = 256 mm × 256 mm, slice thickness = 1 mm, slice spacing = 0 mm, 185 sagittal slices and acquisition time = 6 minutes 32 seconds. Resting‐state functional magnetic resonance imaging (rs‐fMRI) was performed using an echo‐planar imaging (EPI) sequence with the following parameters: TR = 2000 ms, TE = 30 ms, flip angle = 90°, FOV = 240 mm × 240 mm, slice thickness = 4 mm, slice spacing = 0.4 mm, 30 slices acquired in the axial plane, 250 total scans and acquisition time = 8 minutes 26 seconds.

### 2.5 Data preprocessing

Data were analysed using MATLAB R2019a (MathWorks, Natick, MA, USA) with the DPABI (Data Processing & Analysis of Brain Imaging; http://rfmri.org/dpabi) (Yan et al., 2016) and SPM12 (Statistical Parametric Mapping; http://www.fil.ion.ucl.ac.uk/spm) toolkits. The preprocessing steps were as follows:

1. Format Conversion (DICOM to NIFTI): The original rs‐fMRI images in DICOM format were converted to NIFTI format for further processing.
2. Removal of First 10 Time Points: The first 10 time points were discarded to reduce the influence of unstable signals during the initial phase of scanning.
3. Slice Timing: The acquisition times of the 30 slices in each scan (acquired over 240‐time points) were synchronized to the middle slice, which was used as the reference, in order to reduce the influence of signal discrepancies between adjacent slices.
4. Motion Correction (Realign): Involuntary small head movements or minor scanner vibrations during the scanning process may lead to spatial displacement of the images. In this study, participants with horizontal displacement greater than 3.0 mm or rotation greater than 3.0° were excluded.
5. Co‐registration: Each subject's T1 image was segmented into white matter, grey matter and cerebrospinal fluid. The DARTEL (Diffeomorphic Anatomical Registration Through Exponentiated Lie Algebra) method was then used to register and create the optimal template.
6. Spatial Normalization: The optimal template was registered to the standard Montreal Neurological Institute (MNI) space, and the images were resampled to 3 mm × 3 mm × 3 mm isotropic voxel size.
7. Detrending: A linear detrending process was applied to remove any machine‐related drift and subject fatigue effects caused by prolonged scanning.
8. Filtering: A band‐pass filter was applied to remove signals outside the 0.01–0.1 Hz frequency range, which helped reduce noise and isolate relevant neural activity.

### 2.6 Construction of brain functional networks and graphs

The brain's functional network comprises two key components: nodes and edges. In line with previous studies, we employed the GRETNA toolbox and referenced the AAL atlas to segment the brain into 90 regions of interest (45 per hemisphere), which were designated as nodes within the network. The average time series for each node was then extracted, and the Pearson correlation coefficient between pairs of nodes was calculated. These correlation coefficient matrices define the edges of the network, representing functional connections between nodes, resulting in a 90 × 90 connectivity matrix.

A sparsity threshold S was applied to all correlation matrices. S was defined as the ratio of the number of existing edges to the maximum possible number of edges in the network. This approach ensured that all resulting networks had an equivalent number of nodes and edges by applying a subject‐specific correlation coefficient threshold. This normalization minimized potential discrepancies in overall correlation strength between groups, enabling the investigation of differences in relative network organization across groups (Achard & Bullmore, 2007; Zhang et al., 2011). Instead of using a single threshold, a range of sparsity levels was applied to each correlation matrix. The thresholds were determined according to two conditions: (1) The average degree (defined as the number of connections linked to a node) for all nodes in each thresholded network was greater than 2 × log(*N*), where *N* = 90, the number of nodes; and (2) The small‐world coefficient (Sigma) of the thresholded networks was greater than 1.1 for all participants (definition of small‐worldness provided below). The range of sparsity thresholds, 0.05 < S < 0.18, determined through this procedure, ensured that the thresholded networks were compatible with small‐world characteristics (Watts & Strogatz, 1998), while minimizing spurious edges (Achard & Bullmore, 2007; Bassett et al., 2008; He et al., 2008). Within this sparsity range, the largest component sizes of individual networks ranged from 88 to 90. Network analyses were subsequently performed across the small‐world regime (0.05 < S < 0.18) with intervals of 0.01.

### 2.7 Network analysis

#### 2.7.1 Global and node parameters

The global topology of the brain's functional network and the regional characteristics of each node were assessed by calculating both global network parameters and regional node parameters (Rubinov & Sporns, 2010; Wang et al., 2010).

Global parameters included characteristic path length (Lp), clustering coefficient (Cp), normalized clustering coefficient (Gamma), normalized characteristic path length (Lambda), small‐world coefficient (Sigma) and brain network efficiency, which consists of global efficiency (Eglob) and local efficiency (Eloc). Path length refers to the shortest distance between any two nodes in the network and reflects the efficiency of information transfer within the brain. Shorter path lengths typically indicate faster communication between brain regions. Cp (clustering coefficient) measures the tendency of nodes to cluster together within the network. A higher Cp value suggests that nodes are densely interconnected, indicating a more integrated network structure. Elevated Cp, Gamma and Eloc values are indicative of functional segregation in brain networks, referring to the specialization of distinct regions for specific tasks or functions. Conversely, lower Lp, higher Gamma and higher Eglob reflect functional integration, which is the ability of different brain regions to coordinate and collaborate efficiently. Sigma quantifies both functional integration (low Lp) and functional segregation (high Gamma). Functional integration and segregation are fundamental principles in understanding brain network organization. Small‐world networks typically exhibit both regular lattice structures (high Gamma, Gamma > 1) and small path lengths (Lambda ≈ 1), resulting in a Sigma value generally greater than 1.1 (Sigma > 1.1) (Watts & Strogatz, 1998).

For the analysis of nodal parameters, we calculated betweenness centrality, degree centrality (DC), node clustering coefficient (NCp), node characteristic path length (NLp), node efficiency (Ne) and node‐local efficiency (Nle). Betweenness centrality (BC) measures the influence of a node on overall information flow in the network; a higher BC indicates greater traffic passing through the node (Freeman, 1978). Degree centrality (DC) quantifies the number of direct connections a node has with other nodes; a higher DC suggests that the node is more central within the network (Rubinov & Sporns, 2010). The node clustering coefficient (NCp) evaluates the likelihood that neighbouring nodes are interconnected (van den Heuvel & Hulshoff Pol, 2010). Node efficiency (Ne) indicates how efficiently a node facilitates parallel information transmission within the network (Rubinov & Sporns, 2010). Local efficiency (Nle), defined within a subgraph consisting of a node and its neighbours, is considered a measure of network fault tolerance, reflecting how well information is exchanged between neighbouring nodes when the focal node is removed (Achard & Bullmore, 2007).

#### 2.7.2 Modular analysis

**FIGURE 1:**
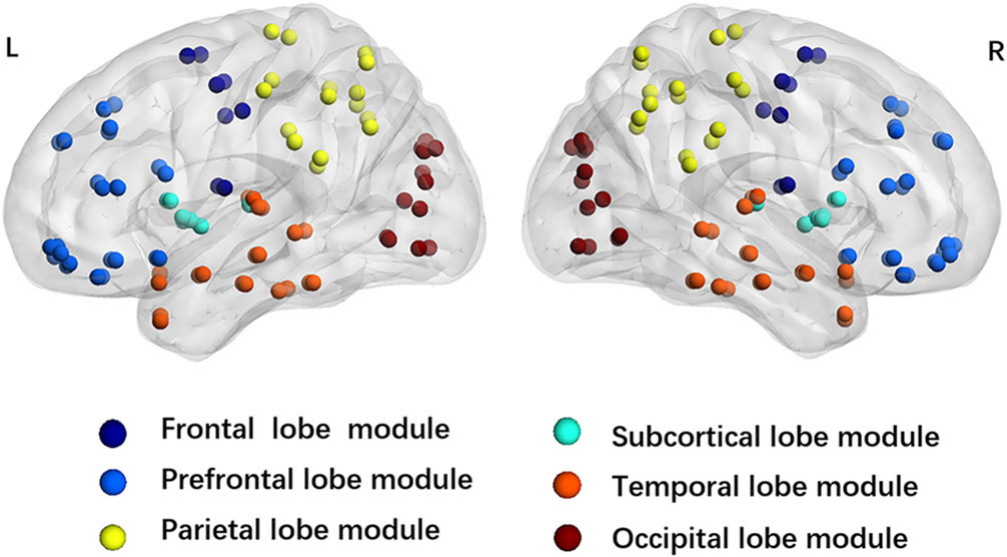
Six modules divided by AAL90 template L:left; R:right.

The AAL90 template divides the 90 regions of interest (ROIs) into six submodules: frontal, prefrontal, parietal, temporal, occipital and subcortical (Figure 1). The mean strength of intra‐ and inter‐module connections was calculated for patients with recent‐onset tinnitus, patients with persistent tinnitus and healthy controls. For each participant, the average intra‐module connection strength represents the mean number of connections within a selected module, while the average inter‐module connection strength reflects the mean number of connections between the selected module and other modules.

### 2.8 Statistical analysis

SPSS 26.0 software was used to analyse the demographic and clinical data of the tinnitus patient groups and the healthy control group. Initially, a chi‐square test was employed to compare gender differences among the three groups and to examine differences in the side of tinnitus between the two patient groups. The Shapiro–Wilk test was subsequently applied to assess the normality of continuous numerical variables across the three groups. For normally distributed continuous variables, including LM, TFI, TFI‐I, TFI‐SC, TFI‐R and TFI‐E, one‐way ANOVA was used for comparisons among the three groups, and two‐sample t‐tests were applied for pairwise comparisons. For non‐normally distributed variables, including age, average hearing threshold, hearing threshold at 8000 Hz, duration, PM, TFI‐C, TFI‐SL, TFI‐A and TFI‐Q, the Kruskal‐Wallis H test was utilized for comparisons among the three groups, and the Mann–Whitney U test was used for pairwise comparisons.

To mitigate the potential impact of arbitrary threshold selection on significant differences in modularity and other network metrics, an integrated network metric was calculated for modularity across predefined thresholds (Achard & Bullmore, 2007; He et al., 2009). Mathematically, the integrated modularity corresponds to the area under the curve (AUC), which was calculated for each network metric. The AUC provides a summarized scalar that represents the topology of the brain's functional connectome, independent of the threshold selection (Zhang et al., 2011). This integrated AUC metric has been previously employed in brain network studies and is sensitive to detecting topological alterations in brain disorders (Achard & Bullmore, 2007). Following this, statistical analysis was performed on the integrated AUC values to assess between‐group differences. A *P*‐value of <0.05 was considered statistically significant. Age, sex, average hearing thresholds and hearing thresholds at 8000 Hz were included as covariates in the analysis of covariance (ANCOVA) or nonparametric tests for the AUC values of all network indicators across the recent‐onset tinnitus, persistent tinnitus and healthy control groups. The least significant difference (LSD) correction was applied as the post hoc test to compare differences between each pair (*P* < 0.05). Bonferroni correction was used for node analysis to reduce Type I errors for simple effects (*P* < 0.05). For module‐wise measures, including intra‐module and inter‐module connectivity, the significance threshold was set at *P* < 0.05, with Bonferroni correction applied for multiple comparisons.

Additionally, Spearman's correlation analysis was conducted using global parameters and nodal parameters that passed the normality test, along with the psychoacoustic characteristics (LM and PM) of tinnitus patients, tinnitus duration, TFI scores and scores from each subscale. The correlation results were adjusted using false discovery rate (FDR) correction for multiple comparisons (*P* < 0.05). A FDR correction at q < 0.05 was applied to correct for multiple comparisons in the correlation analysis.

## 3 RESULTS

### 3.1 General information

**FIGURE 2:**
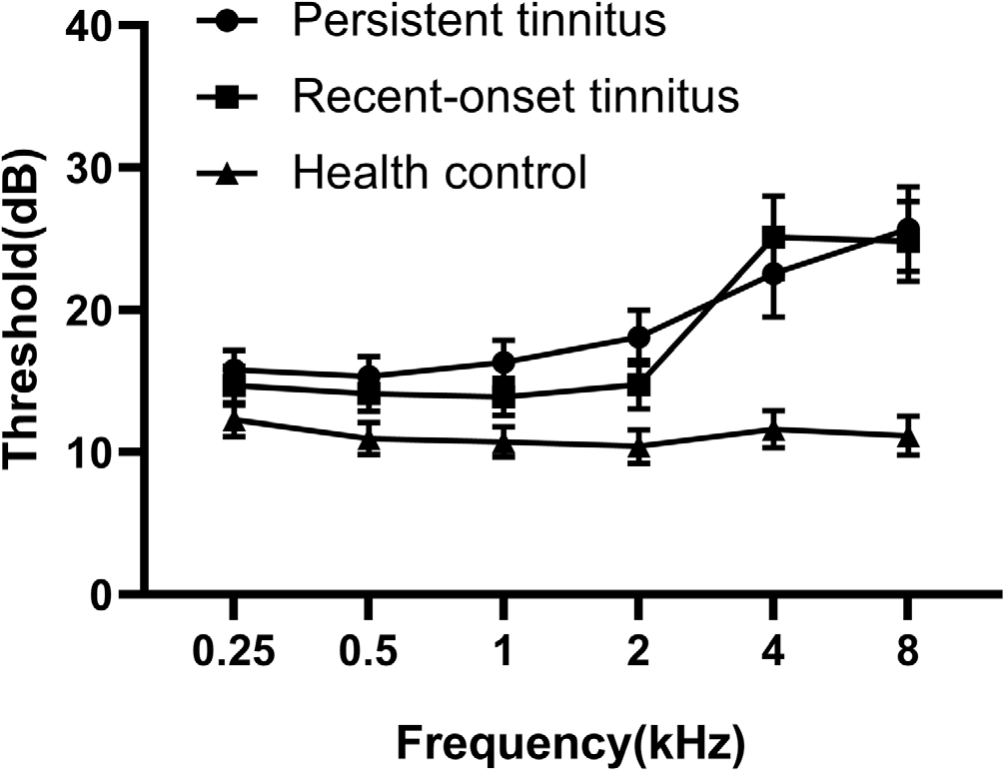
Mean hearing thresholds of the tinnitus patients and health controls. Data are presented as mean ± SEM.

The demographic and clinical data for 28 patients with persistent tinnitus, 23 patients with recent‐onset tinnitus and 27 healthy controls are presented in Table 1. No significant differences were observed in age or gender among the three groups (*P* > 0.05). However, the hearing threshold levels of tinnitus patients were significantly higher than those of the healthy control group (*P* < 0.05), indicating that tinnitus patients had lower hearing thresholds compared to the control group (as shown in Table 1 and Figure 2). Notably, there was a trend suggesting that the TFI scores, as well as all subscale scores, were slightly lower in the persistent tinnitus group compared to the recent‐onset tinnitus group.

**TABLE 1:** Comparison of PT, ROT and HC demographics and clinical characteristics.

	PT (*n* = 28)	ROT (*n* = 23)	HC (*n* = 27)	Statistics	*P*‐value	*P*‐value		
						PT vs. ROT	PT vs. HC	ROT vs. HC
Gender (m/f)	21/7	15/8	17/10	χ^2^ = 1026	0.599			
Age (years)	39.0(32.0, 54.5)	34.0(29.0, 44.0)	40.0(27.0, 54.0)	F = 0.329	0.721	1	1	1
Average HT	15.6(10.0, 24.4)	17.5(12.5, 27.8)	10.8(8.8, 13.8)	F = 5.543	0.006*	1	0.006*	0.005*
HT at 8000 Hz	23.75(13.12,34.37)	25.00(17.50,35.00)	12.50(10.00,15.00)	F = 9.072	<0.001*	1	0.002*	0.001*
Duration of illness (months)	35(12, 60)	2.6(0.5, 3)	NA	Z = ‐6.119	<0.001*	NA	NA	NA
PM (HZ)	5250(2000, 8000)	6000(3000, 8000)	NA	Z = ‐0.310	0.757	NA	NA	NA
LM (dB/HL)	38.4 ± 19.9	44.8 ± 19.0	NA	t = −1.173	0.246	NA	NA	NA
Side (left/right/double)	5/9/14	10/8/5	NA	χ ^2=^5.552	0.062	NA	NA	NA
TFI	36.8 ± 17.3	39.7 ± 11.7	NA	t = −0.698	0.488	NA	NA	NA
TFI‐I	16.4 ± 6.3	16.5 ± 5.3	NA	t = −0.052	0.958	NA	NA	NA
TFI‐SC	13.4 ± 5.7	14.8 ± 4.5	NA	t = −0.926	0.359	NA	NA	NA
TFI‐C	7(1.5, 13)	11(9, 13)	NA	Z = ‐1.817	0.069	NA	NA	NA
TFI‐SL	12(3, 18.5)	13(6, 17)	NA	Z = ‐0.038	0.970	NA	NA	NA
TFI‐A	3(1, 13.5)	8(3, 12)	NA	Z = ‐1.320	0.187	NA	NA	NA
TFI‐R	13.8 ± 8.2	14.1 ± 6.3	NA	t = −0.161	0.872	NA	NA	NA
TFI‐Q	7.5(2.25, 15.5)	12(6, 19)	NA	Z = ‐1.120	0.264	NA	NA	NA
TFI‐E	11.6 ± 7.2	12.5 ± 6.7	NA	t = −0.425	0.673	NA	NA	NA

*Note*: Data with normal distribution are presented as mean ± standard deviation, and data with non‐normal distribution are presented as median (first quartile, third quartile) [M(Q1, Q3)];*P < 0.05; the *P*‐values compared between the two groups are the result of Bonferroni corrected. Abbreviations: PT, Patients with persistent tinnitus; ROT, patients with recent‐onset tinnitus; HC, healthy controls; PM, pitch matching; LM: loudness matching; Average HT: average hearing thresholds (across 500 Hz,1000 Hz,2000 Hz,4000HZ). TFI, Tinnitus Functional Index; TFI‐I, Intrusive Subscale; TFI‐SC, Control Sense Subscale; TFI‐C, Cognition Subscale; TFI‐SL, Sleep Subscale; TFI‐A, Hearing Subscale; TFI‐R, Relaxation Subscale; TFI‐Q, Quality of Life Subscale; TFI‐E, Emotion Subscale; NA, not applicable.

### 3.2 Comparison of global metrics

**FIGURE 3:**
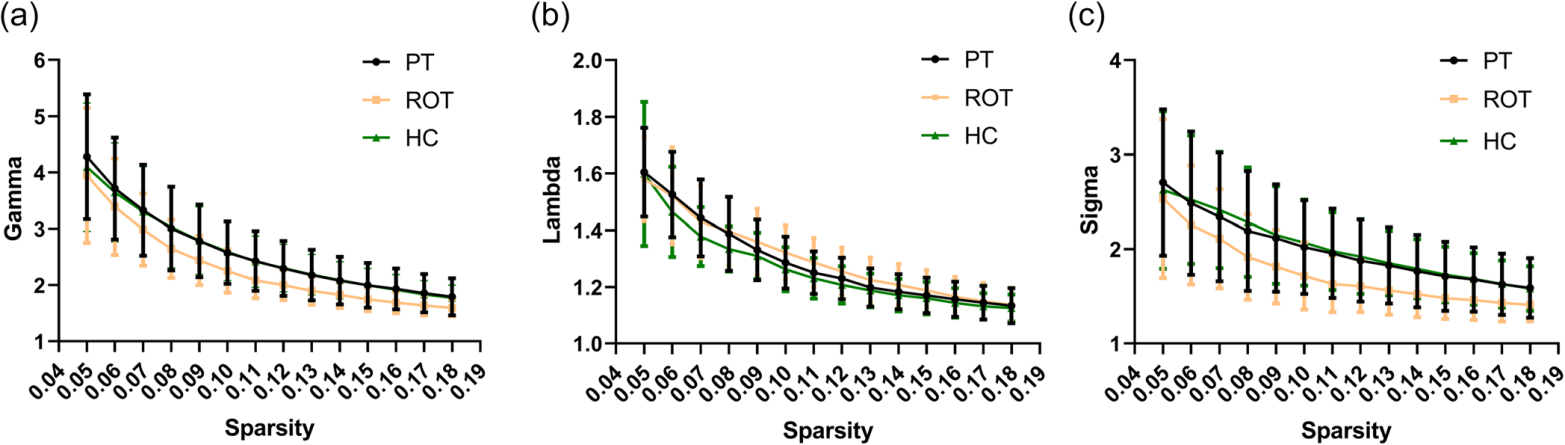
Parameters of the small world in the ROT group, PT group and HC group in the sparsity range of 0.05–0.18. (a) Gamma values of the three groups, (b) lambda values of the three groups, (c) sigma values of the three groups. Abbreviations: PT, persistent tinnitus; ROT, recent‐onset tinnitus; HC, healthy controls; gamma, normalized clustering coefficient; lambda, normalized characteristic path length; sigma, small‐world coefficient.

As illustrated in Figure 3, the recent‐onset tinnitus, persistent tinnitus and healthy control groups all exhibit the typical characteristics of a small‐world network. Specifically, within the sparsity range of 0.05–0.18, the values of Gamma and Sigma were greater than 1, while the values of Lambda were close to 1.

**FIGURE 4:**
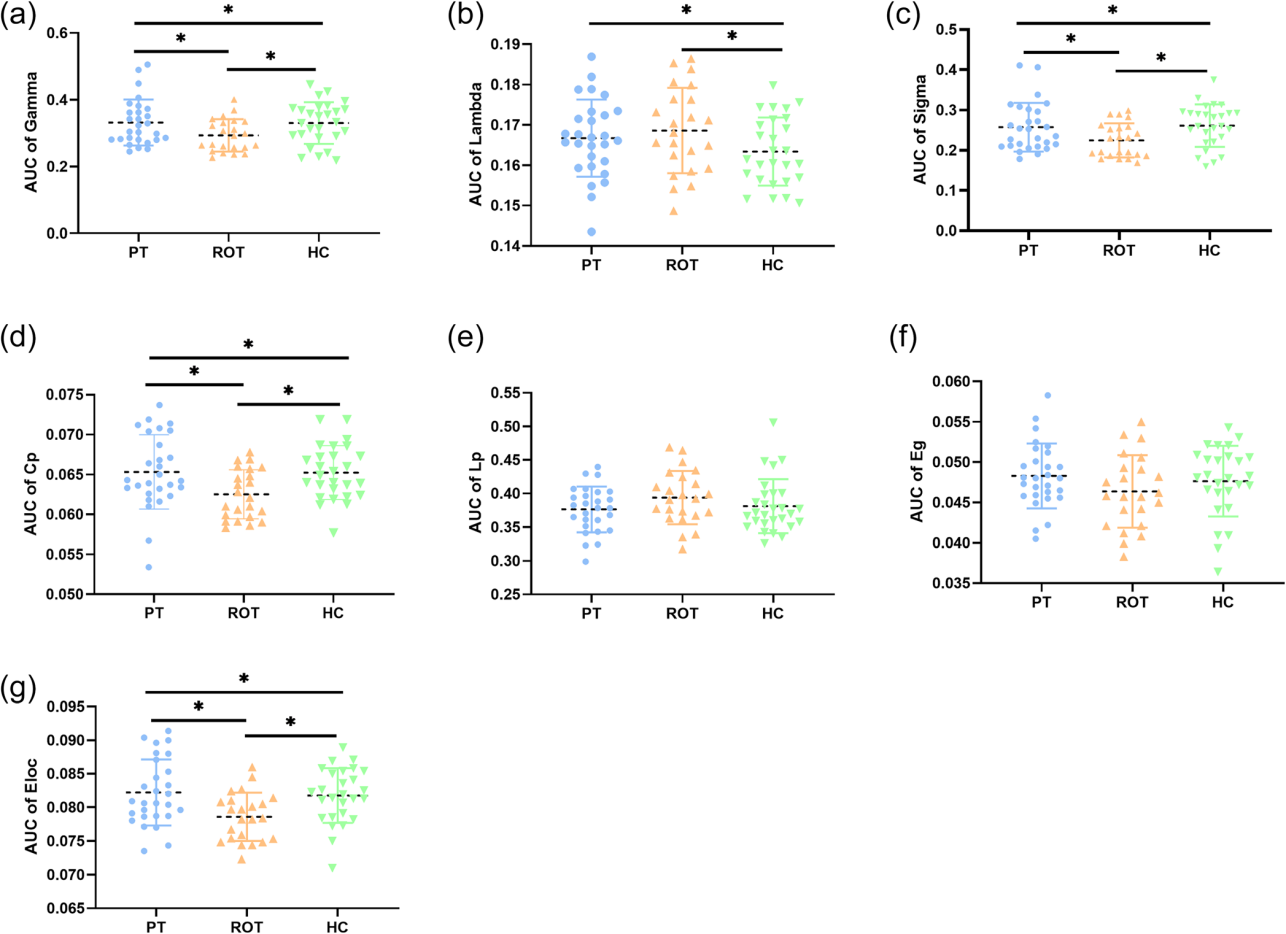
Comparison of global parameters among the ROT, PT and HC groups. (a) Comparison of the AUC of gamma among the three groups; (b) comparison of the AUC of lambda among three groups; (c) comparison of the AUC of sigma among three groups; (d) comparison of the AUC of Cp among three groups; (e) comparison of the AUC of Lp among three groups; (f) comparison of the AUC of Eg among three groups; (g) comparison of the AUC of Eloc among three groups. Abbreviations: ROT, recent‐onset tinnitus; PT, persistent tinnitus; HC, healthy controls; AUC, area under the curve; gamma, normalized clustering coefficient; lambda, normalized characteristic path length; sigma, small‐world coefficient; Lp, characteristic path length; Cp, clustering coefficient; Eglob, global efficiency; Eloc, local efficiency.

Figure 4 and Table 2 present a comparison of global parameter metrics among the recent‐onset tinnitus, persistent tinnitus and healthy control groups. Significant differences were found in the AUC of Gamma, Lambda, Sigma, Cp and Eloc across the three groups. Specifically, the AUC of Gamma, Sigma, Cp and Eloc in the persistent tinnitus group and healthy control group was significantly greater than that in the recent‐onset tinnitus group, while the AUC of Lambda in the recent‐onset tinnitus group was significantly greater than in the healthy control group. No significant differences were observed in the AUC of Lp and Eg among the three groups.

**TABLE 2:** Comparison of global parameters among the ROT, PT and HC groups.

	PT (*n* = 28)	ROT (*n* = 23)	HC (*n* = 27)	Statistics (F)	*P*‐value	*P*‐value		
						PT vs. ROT	PT vs. HC	ROT vs. HC
aGamma	0.332 ± 0.069	0.294 ± 0.049	0.331 ± 0.062	4.491	**0.035**	**0.028**	0.916	**0.038**
aLambda	0.167 ± 0.010	0.169 ± 0.010	0.163 ± 0.008	2.581	**0.044**	0.483	**0.029**	**0.047**
aSigma	0.258 ± 0.061	0.225 ± 0.424	0.261 ± 0.053	3.962	**0.015**	**0.032**	0.793	**0.018**
aCp	0.653 ± 0.005	0.625 ± 0.003	0.652 ± 0.004	4.012	**0.029**	**0.011**	0.945	**0.014**
aLp	0.376 ± 0.033	0.393 ± 0.040	0.382 ± 0.041	1.238	0.302	0.120	0.584	0.303
aEg	0.048 ± 0.004	0.046 ± 0.004	0.048 ± 0.004	1.548	0.197	0.113	0.569	0.299
aEloc	0.082 ± 0.005	0.079 ± 0.004	0.082 ± 0.004	2.543	**0.047**	**0.004**	0.708	**0.010**

*Note*: The *P*‐values compared between the two groups are the result of LSD corrected.

aGamma, Area under the curve for the normalized mean clustering coefficient; aLambda, Area under the curve for the normalized characteristic length; aSigma, area under the curve for the small‐world coefficient; aLp, area under the curve for the characteristic path length; aCp: area under the curve for clustering coefficient; aEg: area under the curve for global efficiency; aEloc: area under the curve for the local efficiency of the network.

### 3.3 Comparison of node metrics

**FIGURE 5:**
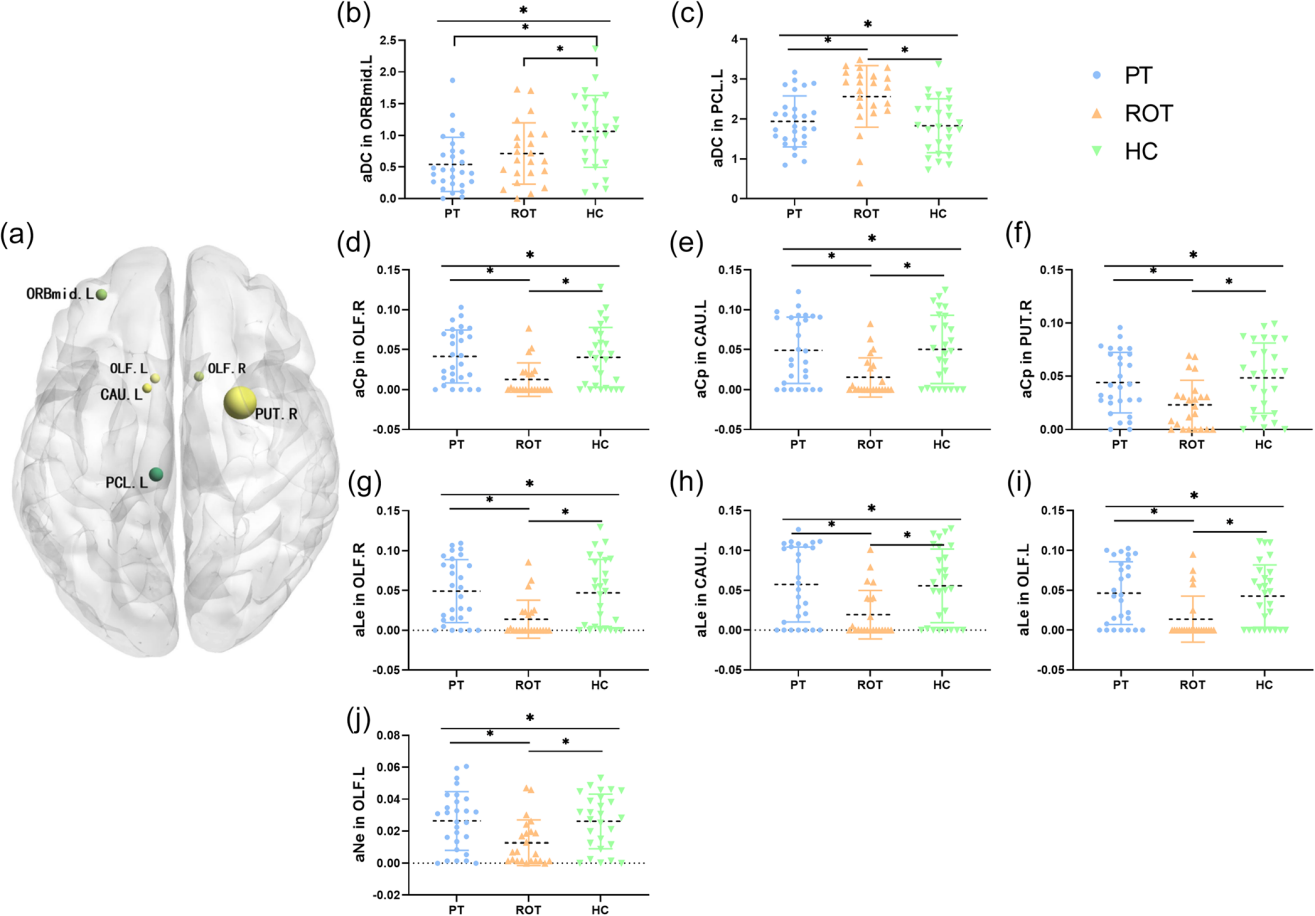
Tested nodes and the AUC of the node parameters. (a) Significant nodes that were statistically different among three groups; (b,c) comparison of the AUC of degree centrality in the left middle frontal gyrus and the left postcentral gyrus among the three groups; (d–f) comparison of the AUC of clustering coefficient in right olfactory cortex, left caudate nucleus and the right lenticular putamen; (g–i) comparison of the AUC of the local efficiency of the node in right olfactory cortex, left caudate nucleus, left olfactory cortex; (j) comparison of the AUC of the nodal efficiency in left olfactory cortex. Abbreviations: aGamma, area under the curve for the normalized mean clustering coefficient; aLambda, area under the curve for the normalized characteristic length; aSigma, area under the curve for the small‐world coefficient; aLp, area under the curve for the characteristic path length; aCp: area under the curve for clustering coefficient; aEg: area under the curve for global efficiency; aEloc: area under the curve for the local efficiency of the network; aDC: area under the curve for the degree centrality of the node; aLe: area under the curve for the local efficiency of the node; aNe: nodal efficiency, ORBmidL: left orbital middle frontal gyrus, OLF.L: left olfactory cortex, OLF.R: right olfactory cortex, CAU.L: left caudate nucleus, PUT.R: right lenticular putamen, PCL.L: left postcentral gyrus.

As depicted in Figure 5 and shown in Tables 3 and 4, after comparing the 90 nodes across the three groups, several significant differences were noted. The AUC of degree centrality (DC) in the left middle frontal gyrus was higher in the healthy control group compared to both the persistent tinnitus and recent‐onset tinnitus groups. In contrast, the AUC of DC in the left postcentral gyrus was higher in the recent‐onset tinnitus group than in both the persistent tinnitus and healthy control groups. Additionally, the AUC of the clustering coefficient (Cp) in the right olfactory cortex, left caudate nucleus and right lenticular putamen was lower in the recent‐onset tinnitus group than in the persistent tinnitus and healthy control groups. The AUC of local efficiency (Ne) in the left olfactory cortex was also lower in the recent‐onset tinnitus group compared to both the persistent tinnitus and healthy control groups.

**TABLE 3:** Location of tested nodes in montreal neurological institute space.

Node		x (mm)	y (mm)	z (mm)
ORBmid.L	Left orbital middle frontal gyrus	−30.65	50.43	−9.62
PCL.L	Left postcentral gyrus.	−7.63	−25.36	70.07
OLF.R	Right olfactory cortex	10.43	15.91	−11.26
CAU.L	Left caudate nucleus	−11.46	11	9.24
PUT.R	Right lenticular putamen	27.78	4.91	2.46
OLF.L	Left olfactory cortex	−8.06	15.05	−11.46

**TABLE 4:** Comparison of node parameters among the ROT, PT and HC groups.

	PT (*n* = 28)	ROT (*n* = 23)	HC (*n* = 27)	Statistics (F)	*P*‐value	*P*‐value		
						PT vs. ROT	PT vs. HC	ROT vs. HC
Degree centrality								
ORBmid.L	0.541 ± 0.429	0.713 ± 0.485	1.064 ± 0.569	4.056	**0.005**	0.544	**0.001**	0.124
PCL.L	1.942 ± 0.640	2.565 ± 0.770	1.830 ± 0.677	5.257	**0.001**	**0.006**	1.000	**0.001**
Clustering coefficient								
OLF.R	0.041 ± 0.033	0.012 ± 0.021	0.040 ± 0.037	3.225	**0.017**	**0.005**	1.000	**0.008**
CAU.L	0.049 ± 0.042	0.015 ± 0.024	0.050 ± 0.043	3.350	**0.014**	**0.006**	1.000	**0.005**
PUT.R	0.044 ± 0.028	0.023 ± 0.023	0.048 ± 0.033	2.809	**0.032**	**0.035**	1.000	**0.008**
Nodal local efficiency								
OLF.L	0.046 ± 0.039	0.014 ± 0.029	0.043 ± 0.039	3.108	**0.020**	**0.007**	1.000	**0.020**
OLF.R	0.049 ± 0.040	0.014 ± 0.024	0.047 ± 0.042	3.566	**0.005**	**0.003**	1.000	**0.006**
CAU.L	0.057 ± 0.057	0.019 ± 0.030	0.056 ± 0.046	3.106	**0.020**	**0.011**	1.000	**0.015**
Nodal efficiency								
OLF.L	0.026 ± 0.018	0.013 ± 0.014	0.026 ± 0.017	3.402	**0.013**	**0.015**	1.000	**0.019**

*Note*: The *P*‐values compared between the two groups are the result of Bonferroni corrected. Abbreviations: PT, persistent tinnitus; ROT, recent‐onset tinnitus; HC, healthy controls; ORBmidL: left orbital middle frontal gyrus, OLF.L: left olfactory cortex, OLF.R: right olfactory cortex, CAU.L: left caudate nucleus, PUT.R: right lenticular putamen, PCL.L: left postcentral gyrus.

### 3.4 Correlation analysis between global and nodal indicators and clinical data

**FIGURE 6:**
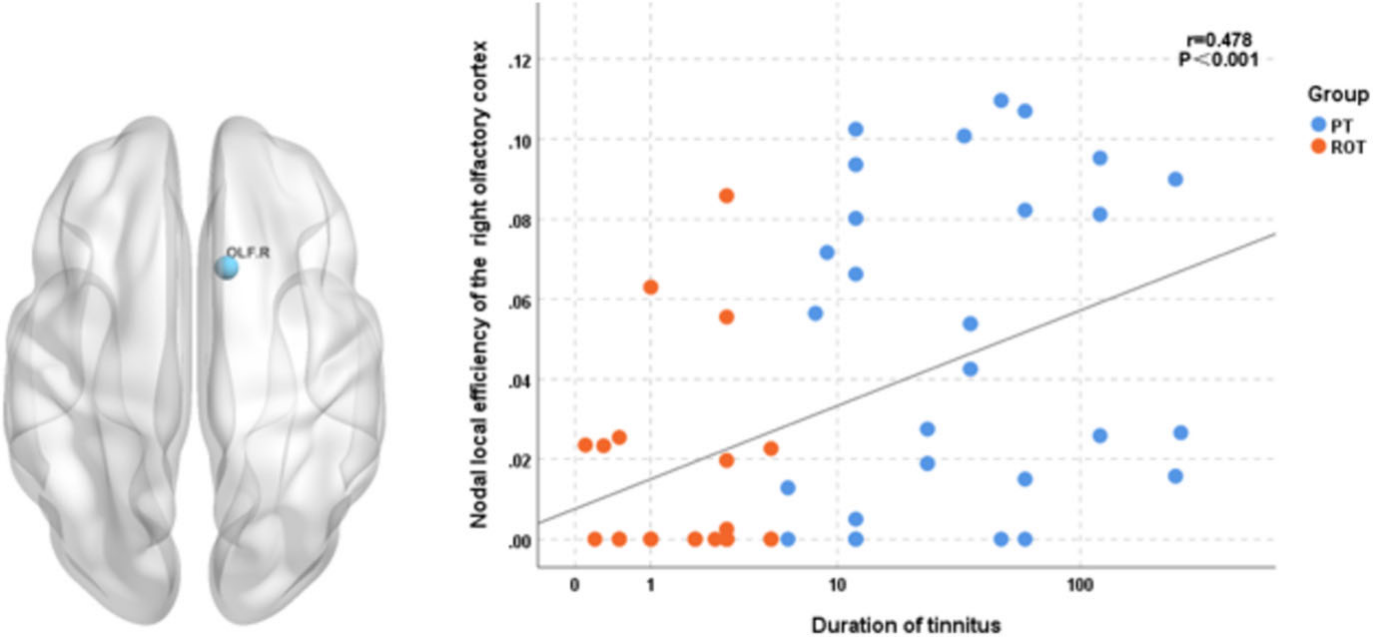
Correlation between nodal local efficiency of the right olfactory cortex and duration of tinnitus. Abbreviations: PT, persistent tinnitus; ROT, recent‐onset tinnitus; HC, healthy controls. OLF.R: right olfactory cortex.

As shown in Figure 6, in the combined tinnitus patient group (51 patients, including both recent‐onset and persistent tinnitus patients), the duration of tinnitus was positively correlated with the nodal local efficiency in the right olfactory cortex (r = 0.292, *P* = 0.037, q < 0.05).

### 3.5 Modular analysis results

**FIGURE 7:**
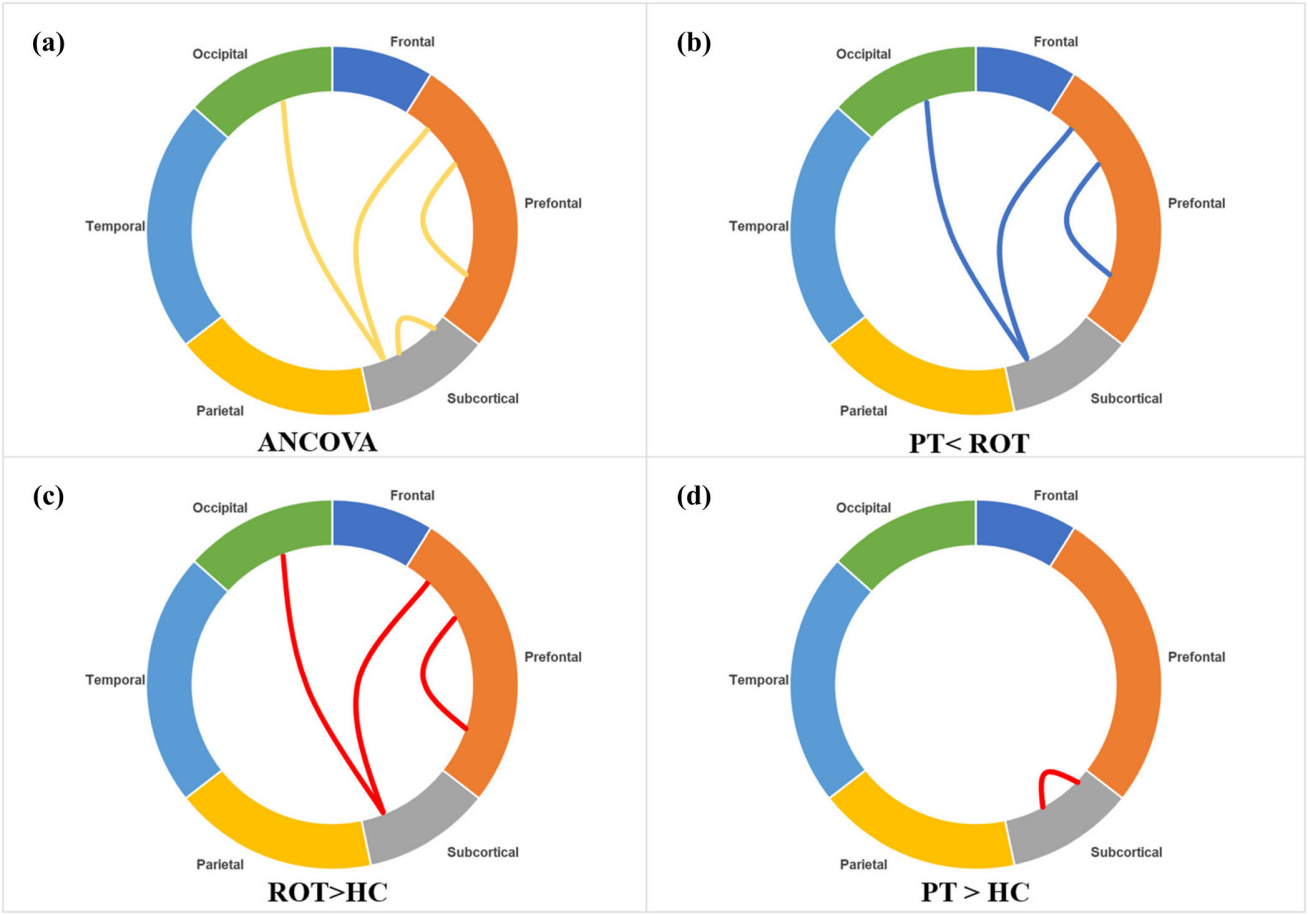
Connections between PT groups, ROT groups and HC groups within and between modules. The functional connectivity strength between the subcortical module, and the occipital lobe module, the prefrontal module and the intra‐frontal module in the PT patients were weaker than those in the ROT group, the intra connectivity strength of the subcortical module was stronger than that in the HC.ANCOVA: covariance analysis. ROT, recent‐onset tinnitus; PT, persistent tinnitus; HC, healthy controls.

The results of the modular analysis indicated significant statistical differences in intra‐network and inter‐network connection strengths among the persistent tinnitus, recent‐onset tinnitus and healthy control groups (Shown in Table 5 and Figure 7). In the recent‐onset tinnitus group, the connection strength between the subcortical module and the occipital module, as well as between the prefrontal module and the prefrontal module, was stronger than in the persistent tinnitus and healthy control groups. Furthermore, the intra‐module connection strength of the subcortical module in the persistent tinnitus group was stronger than that in the healthy control group.

**TABLE 5:** Comparison of intramodule and intermodule connectivity.

Intramodule and intermodule connectivity	Statistics (F)	*P*‐value	*P*‐value		
			PT vs. ROT	PT vs. HC	ROT vs. HC
Frontal‐Prefontal	0.072	0.931	0.780	0.689	0.918
Frontal‐subcortical	1.596	0.210	0.055	0.693	0.124
Frontal–parietal	0.221	0.802	0.536	0.394	0.844
Frontal‐temporal	1.862	0.163	0.078	0.316	0.419
Frontal‐occipital	1.783	0.176	0.772	0.119	0.078
Prefontal‐subcortical	6.571	**0.002**	**0.005**	0.653	**0.002**
Prefontal‐parietal	0.741	0.480	0.790	0.338	0.241
Prefontal‐temporal	2.715	0.073	0.096	0.245	0.580
Prefontal‐occipital	1.818	0.170	0.341	0.489	0.111
Subcortical‐parietal	2.522	0.087	0.261	0.159	0.016
Subcortical‐temporal	0.299	0.742	0.976	0.492	0.533
Subcortical‐occipital	3.480	**0.036**	**0.028**	0.897	**0.039**
Parietal–temporal	2.273	0.110	0.040	0.343	0.250
Parietal‐occipital	3.058	0.053	0.977	0.047	0.055
Temporal‐occipital	0.515	0.600	0.915	0.236	0.218
Frontal	0.099	0.905	0.670	0.922	0.606
Prefontal	4.371	**0.016**	**0.017**	0.765	**0.009**
Subcortical	3.138	**0.049**	0.827	**0.039**	0.080
Parietal	1.858	0.163	0.371	0.069	0.390
Temporal	1.140	0.326	0.317	0.726	0.509
Occipital	2.910	0.061	0.537	0.086	0.026

*Note*: The *P*‐values compared between the two groups are the result of LSD corrected. Abbreviations: PT, persistent tinnitus; ROT, recent‐onset tinnitus; HC, healthy controls.

## 4 DISCUSSION

In this study, graph theory analysis of resting‐state functional magnetic resonance imaging (rs‐fMRI) data was employed to construct the whole‐brain functional network of tinnitus patients at different stages of the disease and healthy controls. Topological differences in the brain functional networks between patients with recent‐onset tinnitus and those with persistent tinnitus were identified. The main findings of this study are as follows: (1) Global parameters, including clustering coefficient (Cp), normalized clustering coefficient (Gamma) and local efficiency (Eloc)—which reflect the functional segregation of brain networks—were lower in the recent‐onset tinnitus (ROT) group compared to the healthy control group, and then increased again in the PT group. (2) Nodal parameters such as nodal clustering coefficient (Ncp) and local efficiency (Nle) in the left caudate nucleus and left olfactory cortex were lower in the ROT group than in the HC group and increased in the PT group. (3) Additionally, enhanced functional connectivity of intermodule connections was observed in the ROT group compared to both the PT and HC groups. These findings not only expand our understanding of the reconfiguration of brain functional connections in tinnitus but also provide evidence for the reorganization of the brain's functional network topology during the chronicization of tinnitus.

Regarding the global properties of functional networks, our results indicate that Cp, Gamma and Eloc were lower in the ROT group than in both the PT and HC groups. However, this difference was not significantly observed in the PT group. The Cp, Gamma and Eloc parameters reflect the functional segregation of brain networks, specifically local interconnectivity (Lv et al., 2018). These results suggest that tinnitus, as a stimulus, may initially disrupt local network communication. As the duration of tinnitus increases, the brain may gradually adapt to this stimulus, leading to a restoration of local network communication. Previous high‐density EEG studies have also shown that, compared to healthy subjects, patients with recent‐onset tinnitus exhibit a significant reduction in frontal cortical activity across all frequency bands, which partially supports our hypothesis (Lan et al., 2021). Furthermore, rs‐fMRI studies utilizing fractional amplitude of low‐frequency fluctuation (fALFF), regional homogeneity (ReHo) and functional connectivity analysis have found reduced neural activity and disconnected connections in areas involving multiple brain regions, particularly in non‐auditory areas, such as the default mode network, attention network, visual network and executive control network, in patients with recent‐onset tinnitus (Zhou et al., 2019).

Additionally, along with the reduction in local interconnectivity, we observed that patients with recent‐onset tinnitus had a decrease in Sigma and an increase in Lambda compared to both the PT group and HC group. Lambda reflects the functional integration of the brain, which refers to the ability of the brain to communicate information globally. Networks with smaller characteristic path lengths (Lp) tend to have higher global efficiency (Eg), suggesting that they are capable of faster communication across the network (Achard & Bullmore, 2007). Our findings align with those of a recent rs‐fMRI study using a graph‐theoretic approach, which reported reduced global efficiency in patients with recent‐onset tinnitus (Lan et al., 2022). The reduced Sigma may indicate an imbalance between the differentiation and integration of brain network functions. We hypothesize that, as the duration of tinnitus progresses, the brain gradually adapts and re‐establishes a balance between functional differentiation and integration within the brain networks.

In terms of node characterization within small‐world networks, our study revealed that several key node parameters, including DC, nodal clustering coefficient (Ncp) and nodal efficiency (Ne), were altered in tinnitus patients with different disease durations compared to healthy controls. These findings indicate node reorganization in the global brain functional network as tinnitus progresses to a chronic state.

DC, which reflects the number of direct connections a node has with the rest of the network (Rubinov & Sporns, 2010), was found to be lower in the left orbital middle frontal gyrus of both PT and recent‐onset tinnitus patients compared to healthy controls. This suggests a potential role for the left orbital middle frontal gyrus in the development of tinnitus. In contrast, DC in the left postcentral gyrus was higher in the ROT group than in the PT and HC groups. The left postcentral gyrus, located in the primary somatosensory cortex, is implicated in the integration of somatosensory and auditory information. Previous research has shown that tinnitus is associated with alterations in somatosensory‐auditory integration and plasticity in the dorsal cochlear nucleus (Sanchez et al., 2007; Shore, 2005). Following cochlear damage and a reduction in auditory nerve input to the cochlear nucleus, somatosensory inputs become upregulated over several days, leading to heightened fusiform cell responses in the dorsal cochlear nucleus to somatosensory stimulation (Shore et al., 2008; Zeng et al., 2009, 2011, 2012). Given this, we speculate that, during the early stages of tinnitus, the left postcentral gyrus may show increased activity due to the upregulation of somatosensory inputs. Over time, however, as tinnitus becomes chronic, the activity in the left middle frontal gyrus decreases, possibly reflecting complex interactions between homeostatic and timing‐dependent plasticity mechanisms.

We also observed that Ncp in the right olfactory cortex, left caudate nucleus and right lenticular putamen was reduced in the ROT group compared to the HC group. Additionally, Nle in both olfactory cortices and the left caudate nucleus was reduced, while Ne in the left olfactory cortex was also lower. Ncp indicates the degree of local connectivity between a node and its neighbouring nodes (Lv et al., 2018), while Nle and Ne measure the node's ability to communicate information with other regions of the brain (Liao et al., 2017). These findings suggest that the efficiency of connection and communication with other regions may be diminished in these areas. The dysfunction observed in the bilateral olfactory cortex may be related to a disruption in the integration of olfactory and auditory information. Previous research has shown that the olfactory cortex receives input not only from the primary auditory cortex but also from the medial geniculate body via retrograde tracking (Wu et al., 2023), which further implicates the olfactory cortex in the pathophysiology of tinnitus. This hypothesis requires further confirmation through cellular physiology and other experimental methods.

Moreover, extensive literature has demonstrated the caudate nucleus's critical role in the onset and progression of tinnitus. The caudate nucleus has been targeted for deep brain stimulation (DBS) in the treatment of new‐onset and refractory tinnitus (Cheung et al., 2019; Perez et al., 2019). Our study provides additional neuroimaging evidence supporting the caudate nucleus as a viable target for tinnitus treatment. Similarly, the putamen, which is part of the striatal network, has been found to be activated during task‐based functional MRI (fMRI) in patients with chronic subjective tinnitus (Wunderlich et al., 2010).

In the correlation analysis, we also observed that the duration of tinnitus was positively correlated with the nodal local efficiency (Nle) in the right olfactory cortex. A study by Haoliang Du's team similarly reported reduced functional connectivity (FC) between the right olfactory cortex and the orbital part of the right middle frontal gyrus, the right precentral gyrus and the left dorsolateral superior frontal gyrus in tinnitus patients (du et al., 2024). The olfactory cortex is a critical component of the limbic system, which plays an essential role in emotional processing, memory formation and sensory perception. It is particularly involved in the processing of emotions related to external stimuli (Li et al., 2013; Lin et al., 2021). Our findings further support the notion that the Nle of the right olfactory cortex may serve as a biomarker for the chronification of tinnitus.

Modular architecture refers to clusters of nodes that are tightly interconnected within local brain regions and sparsely connected to other regions, allowing the brain to balance energy costs and communication efficiency (Sporns & Betzel, 2016). The modular analysis revealed that the connection strength between the subcortical module and both the occipital lobe and prefrontal modules in patients with recent‐onset tinnitus was stronger than in patients with persistent tinnitus and healthy controls. Additionally, the internal connection strength within the prefrontal module was stronger in the recent‐onset tinnitus group. Conversely, patients with persistent tinnitus exhibited stronger internal connectivity within the subcortical module compared to healthy controls. The prefrontal module is known to be involved in cognitive processes as well as emotion regulation (Miller, 2000; Rudebeck & Rich, 2018; Xu, Chen, et al., 2019). fMRI studies have also suggested that deficits in executive function, caused by alterations in the prefrontal cortex, are key factors in the development and persistence of tinnitus (Araneda et al., 2018). Our findings further support the notion that the intra‐prefrontal module connectivity is functionally altered during the onset and maintenance of tinnitus. Furthermore, the strength of the connection between the prefrontal module and the subcortical module was also altered. The subcortical module, which includes neural structures such as the thalamus, caudate nucleus and lenticular nucleus, exhibited reduced local connectivity in the caudate nucleus and lenticular nucleus, as previously noted in the nodal analysis. These changes in modular connectivity are consistent with our earlier findings. We also observed a decrease in connectivity between the prefrontal and occipital modules. rs‐fMRI studies have shown that spontaneous activity and functional connectivity in the occipital lobe are associated with anxiety in healthy adults (Li et al., 2020). Tinnitus patients often report heightened anxiety, which may exacerbate their tinnitus (Pattyn et al., 2016). In patients with recent‐onset tinnitus, the impaired connectivity between the prefrontal and occipital modules could be attributed not only to decreased executive function in the prefrontal cortex but also to tinnitus‐induced anxiety.

In summary, the present study demonstrates changes in the topological properties of brain functional networks during the chronic progression of tinnitus. Specifically, we observed a decline in local network interconnectivity and global information communication ability in patients with recent‐onset tinnitus. Additionally, abnormalities were detected in the function of several key nodes, including the left orbital superior frontal gyrus, left caudate nucleus and bilateral olfactory cortex. These alterations may reflect a central compensatory response to tinnitus.

This study has several limitations. First, the sample size was relatively small, and future studies should aim to expand the sample size to obtain more reliable results. Second, there were differences in the average hearing thresholds between tinnitus patients and healthy controls. Although we endeavoured to control for the impact of hearing loss on the results, future studies should select healthy controls with matched hearing levels. Third, this study was cross‐sectional in nature; thus, longitudinal studies would be valuable to track the reorganization of functional network topological parameters during the chronic progression of tinnitus. Fourth, structural MRI was not analysed in this study; thus, diffusion tensor imaging sequences could be incorporated in future studies to examine structural abnormalities or remodelling of grey and white matter during the chronic phase of tinnitus. Additionally, exploring the relationship between structural and functional network changes would be informative.

## 5 CONCLUSION

In this study, we found that patients with recent‐onset tinnitus exhibited changes in the topology of brain functional networks when compared to healthy controls. However, as the duration of tinnitus increased, most of these changes recovered. These changes included alterations in small‐world properties, reorganization of network transmission efficiency, abnormalities in local node functions and alterations in brain modularity and inter‐module functional connectivity strength.

## AUTHOR CONTRIBUTIONS

Conceptualization: Hui Dai, Yonggang Li and Duo‐duo Tao; Methodology: Shuting Han and Xiaojuan Wu; Software: Shuting Han and Xiaojuan Wu; Validation: Shuting Han, Yongcong Shen and Xiaojuan Wu; Formal analysis: Shuting Han and Yongcong Shen; Investigation: Shuting Han and Yongcong Shen; Resources: Duo‐duo Tao and Jisheng Liu; Data curation: Shuting Han and Yongcong Shen; Writing—original draft preparation: Shuting Han; Writing—review and editing: Shuting Han and Duo‐duo Tao; Visualization: Shuting Han; Supervision: Hui Dai, Yonggang Li and Duo‐duo Tao; Project administration: Duo‐duo Tao; Funding acquisition: Hui Dai, Yonggang Li, Duo‐duo Tao and Jisheng Liu.

## CONFLICT OF INTEREST STATEMENT

The authors declare no conflict of interest.

### PEER REVIEW

The peer review history for this article is available at https://www.webofscience.com/api/gateway/wos/peer-review/10.1111/ejn.16643.

## Data Availability

The datasets used and analysed during the current study are available from the corresponding author upon reasonable request.
